# Post-chemo-radiotherapy response and pseudo-progression evaluation on glioma cell types by multi-parametric magnetic resonance imaging: a prospective study

**DOI:** 10.1186/s12880-023-01135-x

**Published:** 2023-11-06

**Authors:** Maryam Zamanian, Iraj Abedi, Fatemeh Danazadeh, Alireza Amouheidari, Bentolhoda Otroshi Shahreza

**Affiliations:** 1 Department of Medical Physics, School of Medicine, Isfahan University of Medical Sciences, Isfahan, Iran; 2 Department of Radiology, School of Paramedicine, Isfahan University of Medical Sciences, Isfahan, Iran; 3 Department of Oncology, Isfahan Milad Hospital, Isfahan, Iran; 4 Department of Radiology, School of Medicine, Arak University of Medical Sciences, Arak, Iran

**Keywords:** Chemo-radiotherapy, Diffusion-weighted imaging, Glioma, Magnetic resonance imaging, Magnetic resonance spectroscopy, Pseudo-progression

## Abstract

**Background:**

We focused on Differentiated pseudoprogression (PPN) of progression (PN) and the response to radiotherapy (RT) or chemoradiotherapy (CRT) using diffusion and metabolic imaging.

**Methods:**

Seventy-five patients with glioma were included in this prospective study (approved by the Iranian Registry of Clinical Trials (IRCT) (IRCT20230904059352N1) in September 2023). Contrast-enhanced lesion volume (CELV), non-enhanced lesion volume (NELV), necrotic tumor volume (NTV), and quantitative values ​​of apparent diffusion coefficient (ADC) and magnetic resonance spectroscopy (Cho/Cr, Cho/NAA and NAA/Cr) were calculated by a neuroradiologist using a semi-automatic method. All patients were followed at one and six months after CRT.

**Results:**

The results of the study showed statistically significant changes before and six months after RT-CRT for M-CELV in all glioma types (𝑝 < 0.05). In glioma cell types, the changes in M-ADC, M-Cho/Cr, and Cho/NAA indices for PN were incremental and greater for PPN patients. M-NAA/Cr ratio decreased after six months which was significant only on PN for GBM, and Epn (𝑝 < 0.05). A significant difference was observed between diffusion indices, metabolic ratios, and CELV changes after six months in all types (𝑝 < 0.05). None of the patients were suspected PPN one month after treatment. The DWI/ADC indices had higher sensitivity and specificity (98.25% and 96.57%, respectively).

**Conclusion:**

The results of the present study showed that ADC values and Cho/Cr and Cho/NAA ratios can be used to differentiate between patients with PPN and PN, although ADC is more sensitive and specific.

## Introduction

Gliomas, the most common brain tumor, refer to cell types characterized, classified as glioblastoma (GBM), ependymoma (Epn), astrocytoma (Asr), oligodendroglia (Oli), nerve optic glioma, and mixed gliomas [1, 2]. Considering that CRT is a common treatment for brain tumors, assessing tumor cells’ response to radiation is a crucial issue within the treatment plan, as it requires after-care for all types of patients [3, 4].

Despite advances in the field of glioma diagnosis and treatment, the assessment of treatment response remains poor and warrants further investigation [5]. The use of imaging to measure and evaluate tumors is useful for shortening the evaluation time and reducing costs [6]. The criteria for response assessment published by Macdonald et al. in 1990, followed by the Response Assessment in Neuro-Oncology (RANO) working group, were radiological evaluation tools for the treatment of high-grade glioma based on computed tomography (CT) or magnetic resonance imaging (MRI). This criterion, which uses volumetric measurement, is now the standard neuroimaging modality updated in 2010 [3, 7, 8]. Several studies have demonstrated its high efficacy and specificity in the treatment of various glioma-type brain tumors [9–12].

The problem during the treatment of brain tumors is the differentiation progression (PN) of pseudo-progression (PPN), which is probably caused by the increased permeability of tumor vessels and inflammation resulting from radiation therapy, which Temozolomide (TMZ) may aggravate it; thus it is more likely to occur in GBM [13]. The RANO criteria show PPN in increasing contrast enhancement, and studies have reported approximately 36% in high-grade and 20% in low-grade glioma patients [14, 15]. PN and PPN are often confused with radiation necrosis, a focal lesion in the brain caused by radiotherapy. Lesions can have similar imaging manifestations, but usually, PPN subsides without specific treatment, but necrosis that occurs one year after radiotherapy does not subside. Although MRI provides detailed morphological information about tumors, it cannot be used alone [16]. In other words, PN, NPN, and CRT radiation necrosis cannot be distinguished for complete diagnosis, and the need for advanced MRI techniques is essential [17–19]. Quantitative MRI methods, such as diffusion-weighted imaging (DWI), and magnetic resonance spectroscopy imaging (MRSI), have played a very important role in the initial diagnosis and follow-up of patients with possible recurrence [20–24].

Inconsistency in the results of recent studies on changes in quantitative MRI biomarkers after treatment and limitless NPN studies in various glioma types is an important issue that has challenged the power of radiological diagnosis, clarifying the implementation of further studies. In this study, using MRS and DWI indices and based on RANO criteria, the authors sought a suitable method to evaluate the response of all types of glioma cells to treatment.

## Materials and methods

### Study design, inclusion, and Exclusion Criteria

This prospective study was approved in September 2023 by Iran Clinical Trials Registration Organization (IRCT) (IRCT20230904059352N1). The study was conducted on patients suspected of having glioma cell-type tumors who were referred to the oncology department of Isfahan Milad Hospital from November 2021 to June 2022. Based on the type of study design that required patient follow-up and the clinical limitations (e.g., patient migration, patient death, and low-quality imaging data), the sample size was powered by Zhong et al. study [25].

ADC values ​​and three MRS peaks, containing choline (Cho), creatine (Cr), and N-Acetyl Aspartate (NAA), were measured at baseline. In addition, the Contrast-Enhanced Lesion Volume (CELV), Non-Enhanced Lesion Volume (NELV), and Necrotic Tumor Volume (NTV) in all patients were calculated by neuroradiologists using a semi-automated method (Smart Brush - Brain LAB Software). Based on the pathology results following stereotactic surgery, patients were divided into four groups: GBM, Oli, Epn, and Asr, which were treated with RT-CRT.

The criteria for excluding patients from the study were as follow: (I) tumor recurrence and history of RT-CRT treatment; (II) a mixture of types of glioma cells, and other brain tumors; (III) central nervous system (CNS) disorders such as epilepsy and stroke, which probably cause bias in the results of the study; (IV) patients with missing data; and also (V) patients whose total resection of the tumor was not possible either refused surgery or RT-CRT and left the follow-up study due to disease recurrence and the need for special treatment.

MRI-based evaluations were performed one and six months after the completion of RT-CRT for primary acute inflammation. According to the MRI findings and clinical details as well as the RANO criteria, the patients were divided into three categories: non-progression (NPN) with a decrease (~ 35%) or stability of CELV, progression (PN), and pseudo-progression (PPN) with an increase (~ 25% and ~ 50%) in the volume of CELV, respectively. Finally, the sensitivity and specificity of the methods were evaluated using the Receiver Operating Characteristic (ROC) curve [3].

### Chemotherapy and external beam CRT

All selected patients with GBM received a mean total dose of 60 Gy, 2 Gy/fx, and TMZ (75 mg per area of body weight orally daily), followed by adjuvant TMZ (150–200 mg/m2 orally for 5 days during each 28-day cycle) for 6 − 12 cycles depending on the therapeutic response. Patients with Oli, Epn, and Asr disease were subjected to RT alone (mean total dose of 40–60 Gy, 2 Gy/fx).

### MRI protocols

**Fig. 1:**
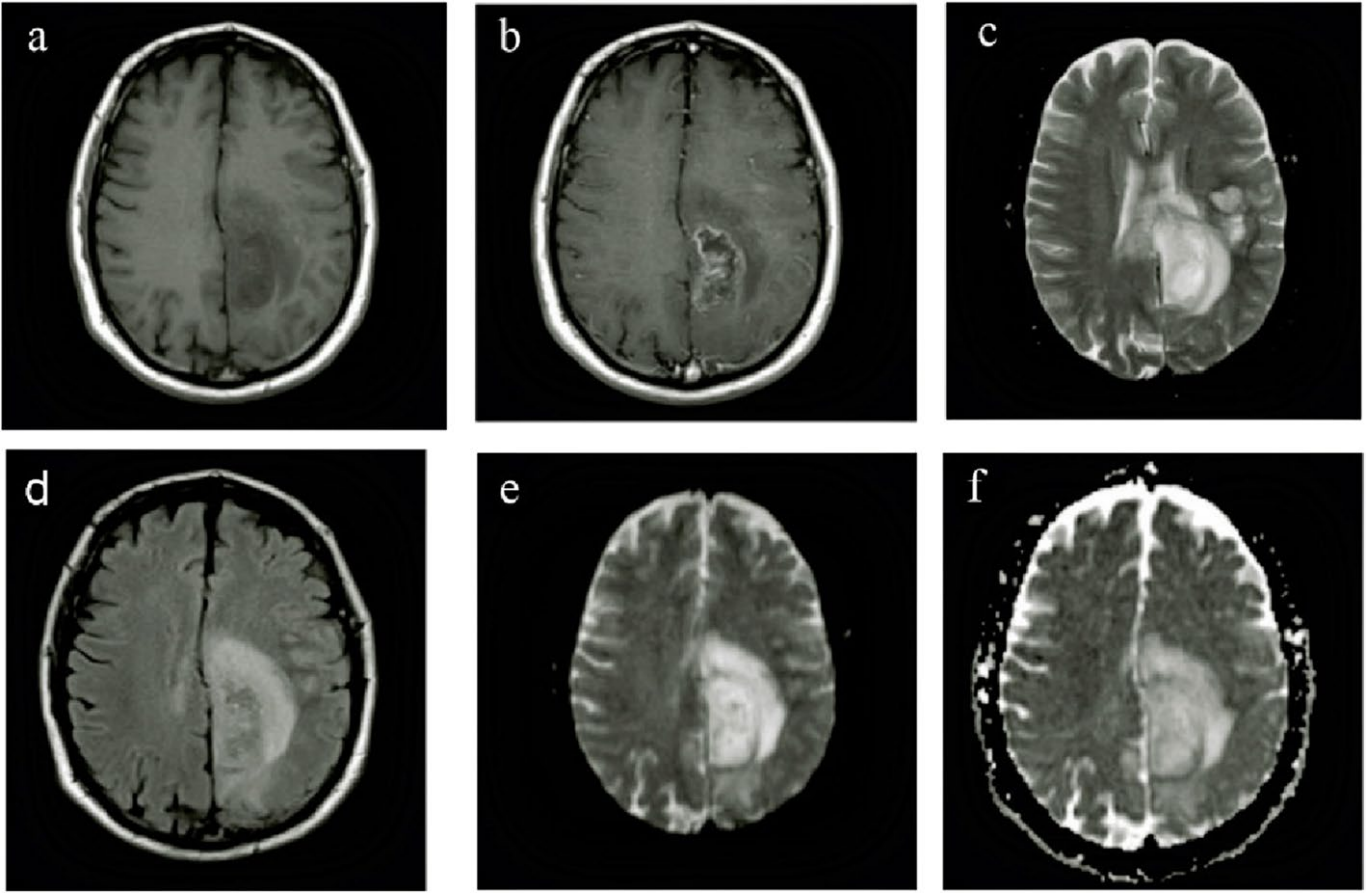
Axial views of a patient with ependymoma at the time of pre-operation: **a** T1-weighted sequences **b** T1-weighted sequences + Gd **c** T2-weighted sequences **d** Fluid-attenuated inversion recovery (FLAIR) sequences **e** Trace, diffusion-weighted imaging (DWI) **f **Apparent diffusion coefficient (ADCmap)

The examinations were performed using a 1.5T MRI scanner (Siemens Healthcare, Erlangen, Germany) equipped with a 20-channel head coil. The MRI protocol consisted of T2-weighted (T2wI) turbo spin echo axial sequences (TSE), with 3200/82 (repetition time ms/echo time ms); 210 mm FOV; 24 slices of 4 mm thickness; 1 mm gap, T1-weighted (T1wI) inversion recovery (IR) sagittal sequences were 2100/19 (repetition time ms/echo time ms), 210 mm FOV; 22 slices of 4 mm thickness; 1 mm gap, fluid-attenuated inversion recovery (FLAIR) coronal sequences were 10,000/120 (repetition time ms/echo time ms), IR delay 2800 ms, (210 × 210 mm) FOV, 28 slices of 4 mm thickness, 1 mm gap (Fig. 1).

### DW imaging (DWI)

**Fig. 2:**
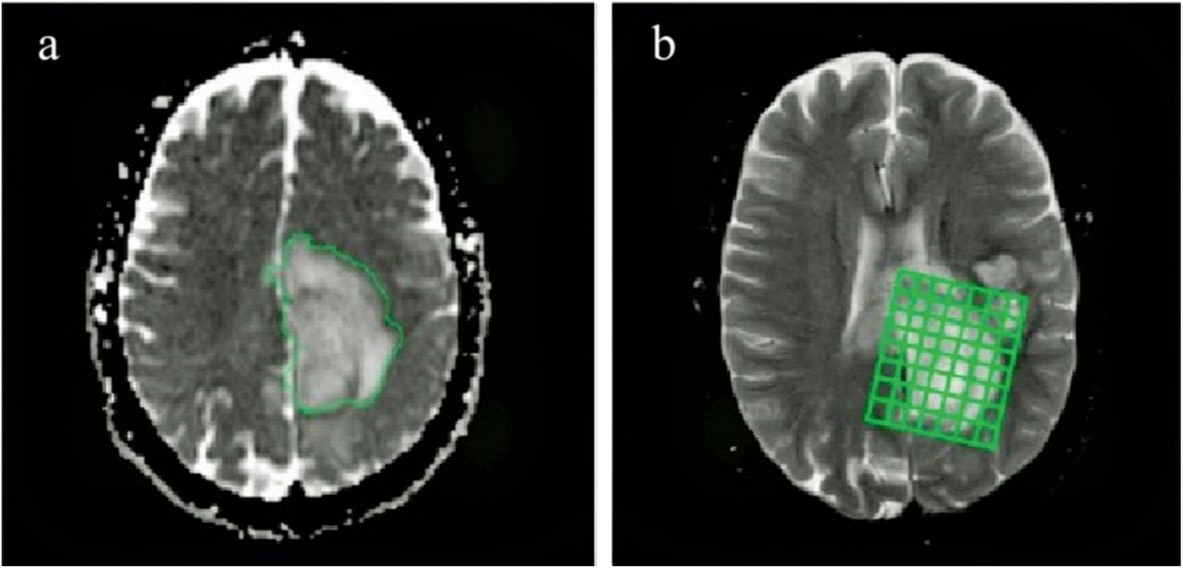
ROI areas drawn in the axial section for quantitative analysis of DWI/ADC values (**a**), as well as the location of Multi-voxel three-dimensional magnetic resonance spectrophotometry imaging (MRSI) indices (Cho, Cr, and NAA) analysis (**b**)

Diffusion images with b values ​​of 0 and 1000 s/mm^2^ were obtained in the transverse plane using a single-shot echo planar imaging (EPI) sequence with diffusion gradient encoding in three orthogonal directions. The parameters for DWI were 4000/84 (repetition time ms/echo time ms), 5 mm slice thickness, 1 mm inter-slice gap, (128 × 128) matrix, (230 × 230 mm^2^) FOV, and one acquisition. ADC values in the solid portion of the tumor using a region of interest (ROI) were calculated using the software manufacturer (Fig. 2). Each ROI was circular or oval and measured of about 40–50 mm^2^ to avoid the mean volume that affects ADC values.

### MRS imaging (MRSI)

Multi-voxel 3D MRS imaging (10 × 10 × 10 mm^3^) was performed on three reference images using a point-resolved spectroscopy pulse sequence (PRESS) prior to contrast injection over the volume of interest (VOI). The VOI was carefully chosen to avoid strong interference from subcutaneous fat and lipids of the skull, and saturation slabs were applied in these areas to further reduce the potential for artifacts (Fig. 2).

Post-processing steps, including frequency shift, baseline correction, phase correction, and peak fitting/analysis using LCModel version 6.3-1 K, were automatically analyzed for the relative signal intensities (areas under the fitted peaks in the time domain) of the following metabolites: Cho, 3.22ppm, Cr, 3.03 ppm, and NAA, 2.02 ppm. Cho/Cr, Cho/NAA, and NAA/Cr ratios were calculated manually. The ROI was positioned corresponding to the voxel of the spectroscopic analysis, using as a reference an axial T2 image for Oli, Epn, and Asr, and an axial enhanced-T1 for GBM. The parameters for MRS were 2100/290 (repetition time ms/echo time ms), (150 × 150 mm^2^) FOV, slice thickness of 10 mm, four acquisition averages, and a scan time 6.82 min.

Finally, T1wI Spin-Echo (SE) was performed after contrast injection, including 10/600 (repetition time ms/echo time ms), (210 × 210 mm^2^) FOV, 23 slices of 4 mm thickness, and 1 mm gap.

### Statistical analysis

The Kruskal-Wallis test was used to examine volume changes (CELV, NTV, and NELV) and imaging indices before, one, and six months after completion of RT-CRT for each type of concussion. Additionally, the Mann-Whitney test and Wilcoxon signed-rank test were used to examine the significance of the relationship between each index and to determine the relationship between the treatment response variables. ROC curves were used to evaluate the sensitivity and specificity of indicators. Analyses were performed using SPSS Version 22 (IBM, Portsmouth, UK), and Origin-pro (Massachusetts, USA, 2023) software. Statistical significance in all tests was considered 𝑝 < 0.05.

## Results

### Patient characteristics

A total of 75 patients (32 females, 43 males) aged 26–69 years (mean 44.8 ± 5.76) with glioma tumor types include GBM (*n* = 35, 46.6%), Oli (*n* = 19, 25.3%), Epn (*n* = 10, 13.33%), and Asr (*n* = 11, 14.66%) precipitated in this study. Based on the results of the immunohistochemistry (IHC) tests, the WHO grade of patients with GBM was found to be type (IV) and other types (II-III). Almost half of the Epn tumors were found near the ventricles, and other types were found in different brain lobes. Demographic details of the patients are presented in Table 1.

**Table 1:** Demographic characteristics and the results of evaluating patients

Glioma type	N	Gender	Age/ Mean ± SD	Grade	Location	PN (1 m)	PPN (1 m)	PN (6 m)	PPN (6 m)
**GBM**	35	25 M-10 F	28–66 (48.08 ± 10.8)	(35)V	Fron > Temp > Occi ~ Part	(*n* = 7) 20%	**(** ***n*** ** = 0) 0%**	**(** ***n*** ** = 21) 60%**	**(** ***n*** ** = 9) 25%**
**Oli.**	19	9 M-10 F	30–54 (41.0 ± 6.7)	(13)II(6)III	Fron > Temp ~ Part	(*n* = 4) 21.05%	(*n* = 0) 0%	(*n* = 8) 42.1%	(*n* = 3) 15%
**Epn.**	10	6 M-4 F	6–40 (28.1 ± 12.9)	(7)II (3)III	Vert > Fron ~ Temp	**(** ***n*** ** = 3) 30%**	(*n* = 0) 0%	(*n* = 4) 40%	(*n* = 2) 20%
**Asr.**	11	6 M-5 F	17–69 (41 ± 13.89)	(8)II (3)III	Fron ~ Temp > Part	(n = 3) 27.2%	(*n* = 0) 0%	(*n* = 5) 45.4%	(*n* = 2) 18%

*GBM* Glioblastoma, *Oli* Oligodendroglia, *Epn* Ependymoma, *Asr* Astrocytoma, *Fron* Frontal, *Temp* Temporal, *Occi* Occipital, *Part* Partial, *PN *Progression, *PPN* Pseudo-progression, *m* Month, *N* Number, *SD* Standard deviation

### Volumes change

The mean CELV, NTV, and NELV before and after RT-CRT, as well as the statistically significant levels for all three time steps of any specific glioma, are explained in Table 2. Generally, a reduction in M-CELV was observed in the number of GBM (29), Oli (14), Epn (8), Asr (8); and GBM (6), Oli (7), Epn (2), Asr (2) patients after one and six months of CRT, respectively.

**Table 2:** The mean volume of the tumor after surgery and post-RT-CRT and its changes

Mean ± SD^1^	CELV (cm^3^)				NTV (cm^3^)				NELV (cm^3^)			
	Pre-RT-CRT	1 m Post-CRT	6 m Post-CRT	p-v	Pre-RT-CRT	1 m Post-CRT	6 m Post-CRT	p-v	Pre-RT-CRT	1 m Post-CRT	6 m Post-CRT	p-v
**GBM.**	61.7 ± 1.1	43.4 ± 1.2	63.1 ± 0.8	*	42.2 ± 0.8	37.2 ± 0.6	45.9 ± 1.1	*	51.2 ± 1.5	39.6 ± 1.2	43.03 ± 21.1	*
**Oli.**	54.6 ± 1.8	40.4 ± 0.9	45.4 ± 1.1	*	26.8 ± 1.3	22.7 ± 1.7	22.6 ± 1.1	*	24.8 ± 0.8	15.5 ± 1.1	14.7 ± 0.7	*
**Epn.**	46.3 ± 0.4	30.7 ± 0.9	36.5 ± 1.0	*	30.6 ± 1.0	23.6 ± 0.7	22.3 ± 0.9	*	26.2 ± 0.7	21.6 ± 0.9	24.4 ± 1.0	*
**Asr.**	52.3 ± 0.4	47.7 ± 0.7	48.3 ± 0.9	*	29.4 ± 0.9	31.7 ± 0.7	36.4 ± 0.6	*	12.6 ± 1.0	11.7 ± 1.2	13.4 ± 0.9	*

Confidence Interval = CI (0.95), p-v = *p*-value (* = (𝑝 < 0.05), ** = (𝑝 > 0.05))

### DWI index change

Patient follow-up showed a decrease in M-ADC at one month and an increase at six months, although the changes were reduced specifically in patients with PN. The M-ADC changes and CELV in six months showed a significant difference in all types and also showed a significant difference between PN, and NPN within M-CELV (𝑝 = 0.0112), M-NELV (𝑝 = 0.0178), and M-NTV (𝑝 = 0.002). Significantly higher changes were observed within M-CELV and NELV for PN vs. NPN. The highest percentage of incremental change was observed between one and six months in patients with GBM. The relationship between M-ADC changes and CELV was significant only between before and six months after treatment (𝑝 < 0.05).

**Fig. 3:**
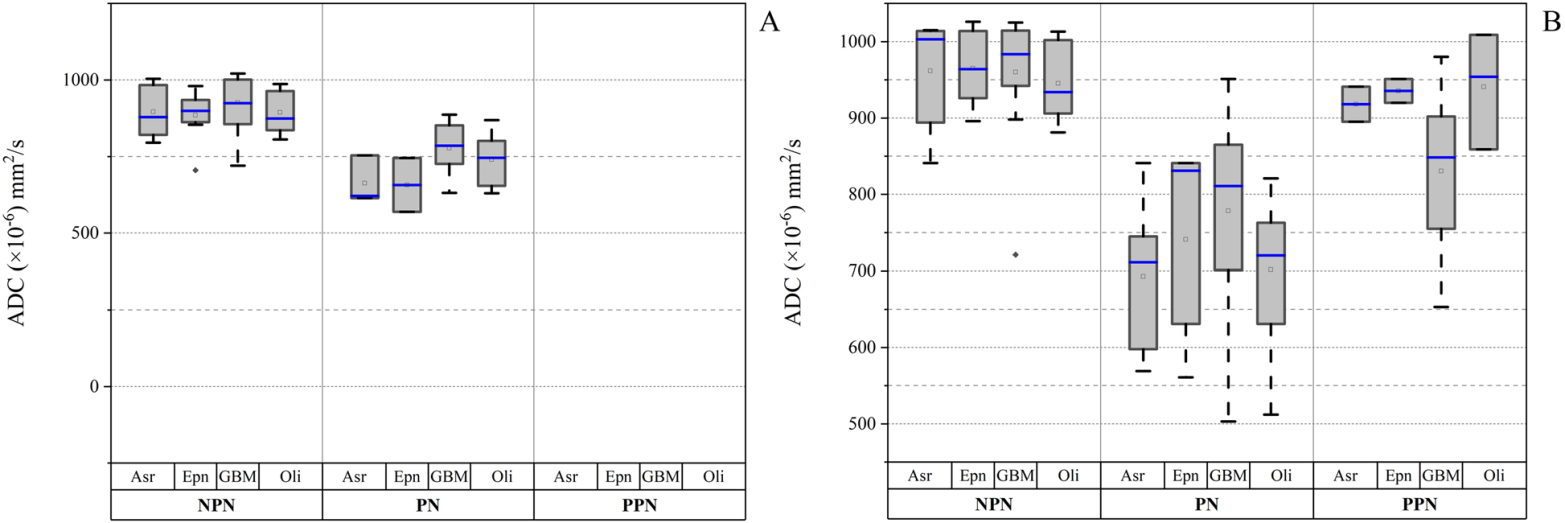
The changes in the mean-ADC (M-ADC) ×10^−6^ values, one-month (**A**) to six months (**B**) after RT-CRT, separately for progressions (PN), non-progression (NPN) and pseudo-progression (PPN) for glioma cancer types including: glioblastomas (GBM), oligodendroglia (Oli), ependymoma (Epn), and astrocytoma (Asr)

The distribution of the Mean-ADC index (M-ADC×10^−6^ mm^2^/s) one and six months after RT-CRT in glioma types is presented in Fig. 3. The ADC index of PPN patients had higher values than PN (𝑝 = 0.031).

### MRS indices change

In the longitudinal analysis of MRS metabolites in glioma types, the mean concentration of Cr (M-Cr) was almost constant (2.3 ± 0.65 M-Cr) during one to six months. Total M-NAA and Cho levels decreased during follow-up, although an increase was observed in PN patients. However, M-Cho/Cr and Cho/NAA increased, and M-NAA/Cr decreased in patients with PN. Also, no significant difference was detected in M-NAA/Cr ratios between all glioma types (𝑝 > 0.05). There was a significant difference between the M-Cho/Cr, Cho/NAA, and NAA/Cr changes and six months M-ADC and the CELV changes in all types of glioma (𝑝 < 0.05).

The mean changes of Cho/Cr, Cho/NAA, and NAA/Cr ratios on PN patients before and six months after RT-CRT were for GBM (0.23 ± 0.47), (0.09 ± 0.46), and (-0.84 ± 0.1), for Oli, were (0.15 ± 0.38), (0.12 ± 0.66), and (-0.1 ± 0.18), for Epn were (0.15 ± 0.55), (-0.09 ± 0.17), and (0.11 ± 0.21), for Asr were (0.07 ± 0.09), (0.05 ± 0.1), and (-0.02 ± 0.15), respectively. In addition, the mean changes of Cho/Cr, Cho/NAA, and NAA/Cr ratios on PPN patients before and six months after RT-CRT were for GBM (0.41 ± 0.31), (0.21 ± 0.74), and (0.02 ± 0.08), for Oli, were (0.26 ± 0.21), (0.5 ± 0.05), and (-0.24 ± 0.05), for Epn were (-0.39 ± 0.03), (-0.21 ± 0.31), and (-0.02 ± 0.1), for Asr were (0.17 ± 0.06), (0.1 ± 0.26)), and (-0.06 ± 0.10), respectively.

**Fig. 4:**
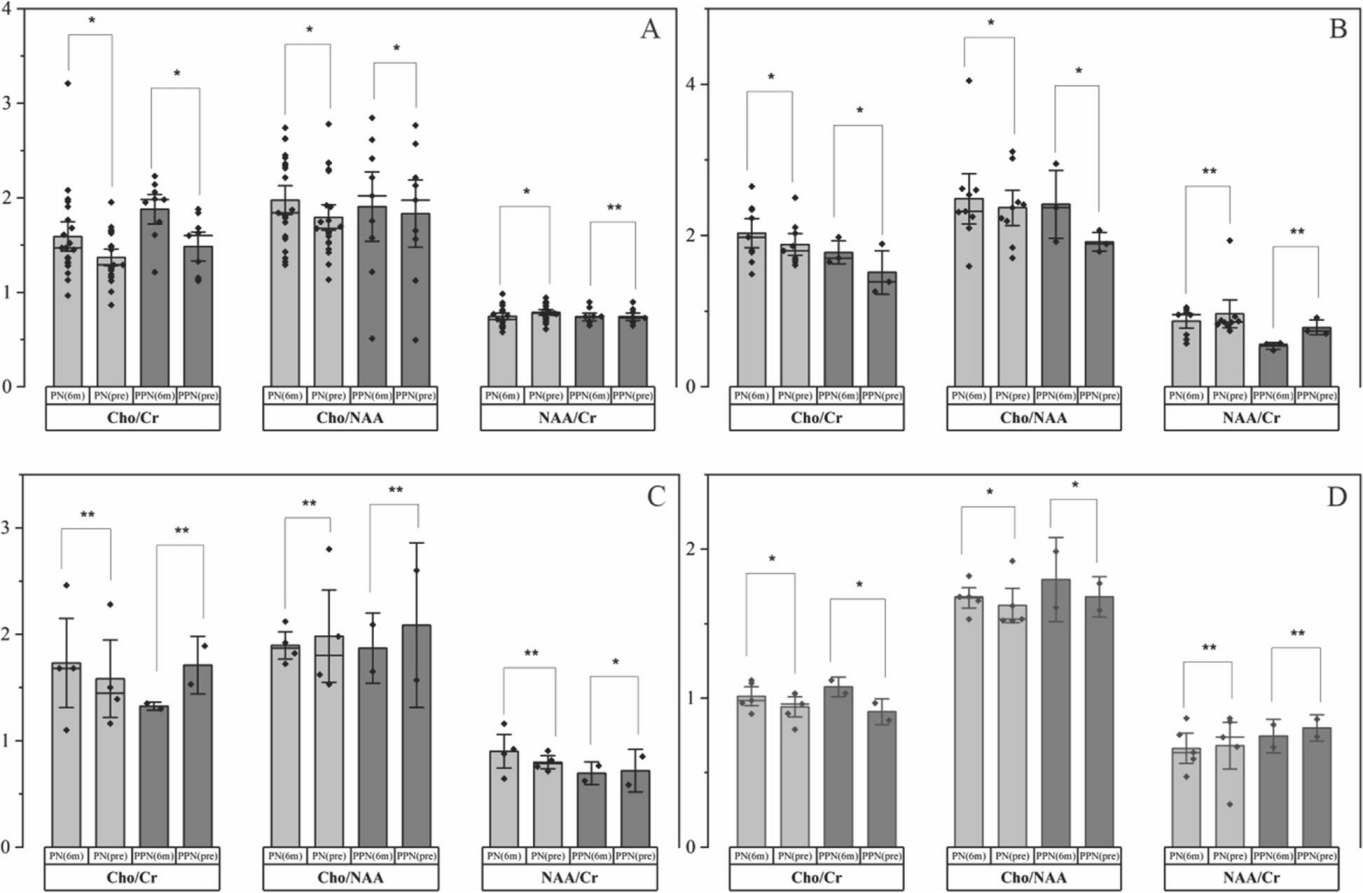
Metabolic evaluation ratios pre- and six months after RT-CRT for PN and PPN patients (* = (𝑝 < 0.05), ** = (𝑝 > 0.05), **A** = GBM, **B** = Oli, **C** = Epn, **D** = Asr)

The M-Cho/Cr, Cho/NAA, and NAA/Cr ratios and significant evaluations for PN and PPN patients before and six months after RT-CRT in all glioma types are presented in Fig. 4. The incremental change of Cho/Cr and Cho/NAA for all types of glioma (except PN, and PPN patients on Epn) was incremental and significant for PN and PPN patients. This increase in the ratio for PPN was somewhat higher than for PN patients (𝑝 < 0.05). The NAA/Cr ratio was mostly decreasing or slightly increasing in all types of glioma, which decrease was significant only in GBM PN and Epn PPN patients (𝑝 < 0.05).

### Use of TMZ evaluation

**Fig. 5:**
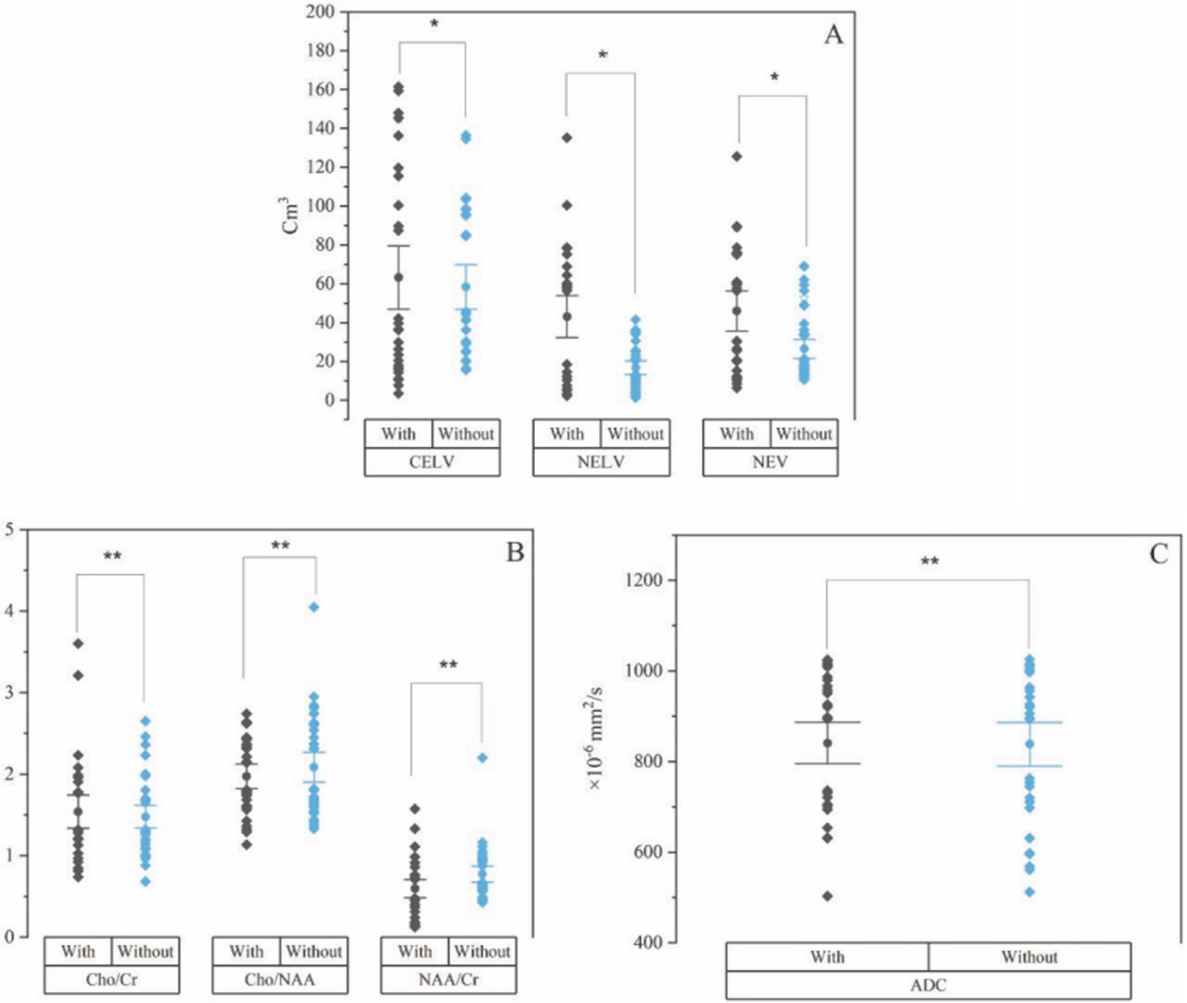
Calculated values regarding the relationship between the use of TMZ and volumes (CELV, NTV, and NELV) and indicators of quantitative MRI and the statistical significance level (**a** = volumes, **b** = MRS, and **c** = ADC, * = (𝑝 < 0.05), ** = (𝑝 > 0.05)

The results of the evaluation of the relationship between the use of TMZ and volume change (CELV, NTV, and NELV) and indicators of quantitative MRI after six months are presented in Fig. 5. A significant relationship was observed only for all volumes when TMZ was used.

### Sensitivity and specificity evaluation

**Fig. 6:**
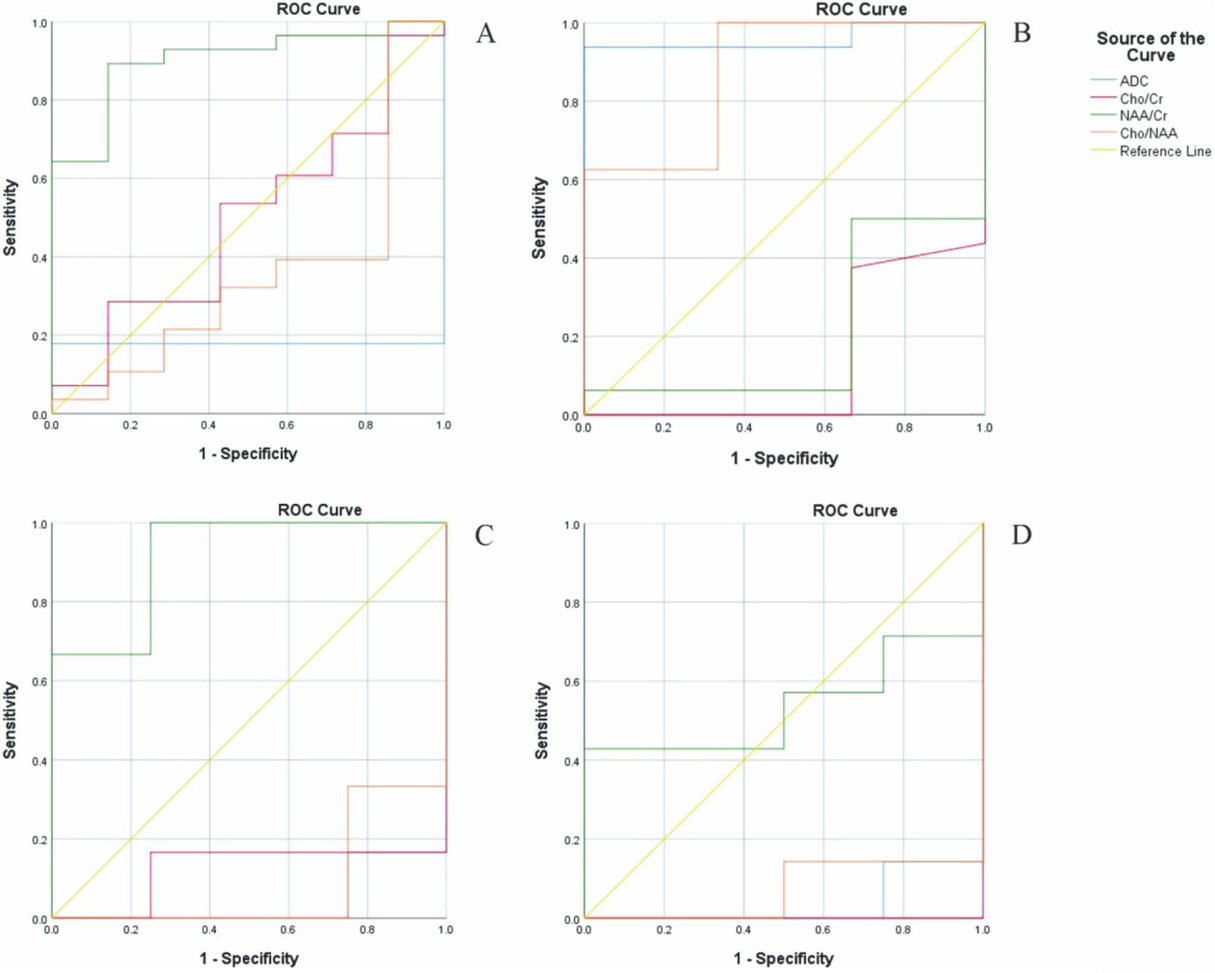
ROC curve of ADC (A), Cho/Cr (B), Cho/NAA (C), NAA/Cr (D) ratios, using a univariate logistic regression model

The sensitivity and specificity of the diffusion and metabolic imaging indices according to the number of correct and incorrect diagnoses, based on the results of the RANO criteria, are presented in Table 3. PN at one and six months after RT-CRT follow-up of the patients was, approximately18.66% and 44%, and NPN was 0% and 21.3%, respectively. The ROC curves for Cho/Cr, Cho/NAA, NAA/Cr ratios, and ADC values are presented in Fig. 6.

**Table 3:** Sensitivity, and specificity of DWI, and MRS indices for Glioma cell types

		GBM				Oli.				Epn.				Asr.			
		Sens	Spec	AUC	Sig.	Sens	Spec	AUC	Sig.	Sens	Spec	AUC	Sig.	Sens	Spec	AUC	Sig.
**DWI** **(%)**	**ADC**	98.3	92.4	0.179	*	100	95.5	0.958	*	98.4	100	0.042	*	96.3	98.4	0.036	*
**MRS** **(%)**	**Cho/Cr**	87.1	85.7	0.495	**	80.6	75.9	0.135	*	85.4	87.1	0.125	**	90.6	87.3	0.000	*
	**NAA/Cr**	82.1	86.9	0.903	*	79.6	82.4	0.208	**	87.1	88.9	0.917	*	82.4	84.6	0.536	**
	**Cho/NAA**	89.6	88.4	0.352	**	84.6	87.2	0.875	*	84.3	86.7	0.083	*	81.3	83.1	0.071	*

*Sens* Sensitivity, *Spec* Specificity, *sig* significant level, * = (𝑝 < 0.05), ** = (𝑝 > 0.05)

## Discussion

This prospective study was conducted to evaluate the use of quantitative MRI methods in conjunction with RANO criteria for assessing the response to RT-CRT and PPN in different types of glioma. Therefore, quantitative measurements such as CELV, NELV, NTV, ADC, and MRS metabolite ratios, were calculated before and (one and six) months after RT-CRT.

After one month, the highest PN occurred in Asr, any PPN was reported in all glioma cell types, and the highest most number of PN and PPN after six months was related to GBM (Table 1). According to volume changes at one month after RT-CRT, which indicates the greatest effect of treatment, the maximum reduction of CELV and NELV was observed in GBM and NTV in Epn, and the maximum therapeutic effect of CRT (GBM) was observed in M-CELV (Table 2). However, the therapeutic effect was not observed based on volume changes between one and six months (𝑝 < 0.05).

The total reduction in the M-ADC observed for PN after one and six months likely indicates relapse in all types of glioma cells; however, this reduction in patients with PPN was lower and M-ADC was higher in these patients (Fig. 3). Based on the significant level of changes in CELV and ADC only in the two time steps before and six months after the RT-CRT is in the same manner (𝑝 < 0.05).

The total changes in mean metabolic ratios increased for M-Cho/Cr, in a decreasing or constant manner for M-NAA/Cr, and different results for the Cho/NAA ratio in glioma cell types are shown. In patients with PN, there was a decrease in the Cho/Cr ratio in all types of glioma, a decrease in Cho/NAA, and an increase in NAA/Cr, except in Epn patients. In addition, in patients with PPN, the changes decreased in the Cho/Cr and Cho/NAA ratios in all types except for Epn, and the NAA/Cr ratio increased in all types. It seems that the changes in the metabolic ratios in patients with Epn are almost in contrast to those in other types of glioma (Fig. 4).

Considering the relationship between all ratios in glioma cell types and six months’ changes in CELV, metabolic changes are also a suitable factor for predicting the response to treatment. The relationship between the changes in these ratios and ADC is in the same direction.

The best index in evaluating all types of glioma cells was ADC as 98.3%, 100%, 100% and 96.3% for GBM, Oli, Epn and Asr (𝑝 < 0.05). Among the metabolic ratios, the highest sensitivity was on GBM (NAA/Cr = 82.1%), Oli (Cho/NAA = 84.6%), Epn (NAA/Cr = 87.1%) and Asr (Cho/Cr = 90.6%), (𝑝 < 0.05) (Table 3).

The effect of TMZ on therapeutic volumes was observed only on NTV and NELV, but had no effect on diffusion and metabolic results; however, a significant effect was observed in patients with PPN (Fig. 5).

Most studies have been performed only on the GBM type. By examining 49 GBM, claims were based on the highest correlation between the M-ADC and M-CELV (*p* = 0.0112 vs. *p* = 0.0144) and NELV in patients with PPN [26].

The studies (on 22 to 68 GBM), similar to the present study, observed higher ADC values in PPN patients and a study on PN (24 patients) showed a decrease in ADC compared between pre-RT and post-one month, and one study (50 children) observed a greater decrease in ADC changes for PN compared to PPN patients [27–31]. However, the contradiction in the results of studies regarding ADC is evident, in such a way that in one study for PN patients (30 patients) it is decreasing, and in another study it is increasing for both patients with PN and NPN (10 patients) [32, 33]. In a study (21 children) there was no significant difference between the average ADC and the tumor volume for PPN, and one study (50 children) observed a greater decrease in ADC changes for PN than for patients with PPN [31, 34].

The studies (between 22 and 68 GBM), similar to the present study, observed higher ADC values in PPN patients [27–29]. However, a significant difference was observed in the results of the studies: in the study (21 children), there was no significant difference between the mean ADC and the tumor volume for PPN, and even one study (50 children) observed a decrease in ADC changes in patients with PPN [31, 34].

Regarding metabolic ratios, a general increase in the concentration of Cho and NAA and the stability of Cho in one study (16 patients), and an increased change in Cho and Cho/Cr in PN patients in another study, while there was no significant difference between the changes in Cho and NNA in the study [35, 36]. To differentiate PPN from PN by metabolic evaluation, higher values obtained for the ratio of Cho/NAA and Cho/Cr were contrary to the studies on GBM patients, who observed a lower amount [37, 38].

In the simultaneous evaluation of several types of glioma cells (10 glioma patients including GBM grade IV, one Oli, and one Asr both grade II), despite the decrease in the metabolic concentrations of NAA and Cho/NAA in all patients, the concentration of Cho/NAA increased in PN patients, although the data analysis confirmed the changes in ratios in PN patients in the studies [30, 32, 39].

Furthermore, the results of the study showed the absence of a significant difference between MRS ratios and ADC [32]. This relationship was confirmed by the results of another study (80 patients) in relation to different degrees of glioma [40].

In addition, differences in the sensitivity and specificity of MRI indices were observed. The higher sensitivity and specificity of DWI compared to MRS imaging has been confirmed in other studies. In a study by Meissner et al., Cho/Cr in Asr (90.6%), NAA/Cr in Epn (87.1%), and Cho/NAA in GBM (89.6%) were the most sensitive, and Cho/Cr in Epn and Asr (87.1%), NAA/Cr in Epn (88.9%), and Cho/NAA in GBM (89.9%) had the highest specificity [39]. In in a study by Bulik et al., both sensitivity and specificity of ADC were estimated to be 100%; the ratios of NAA/Cr for both PN, and PPN was (94.4% and 91.7%), and Cho/NAA was (100% and 91.7%), GBM with a statistically significant level (p < 0.05) [30]. Lutomolo et al. reported that the sensitivity and specificity of different ratios of MRS in high-grade glioma were much higher than in low-grade glioma (Cho/Cr: 80% vs. 33.3%, NAA/Cr: 83.3% vs. 54.5%, Cho/NAA: 84.6% vs. 80%), (Cho/Cr: 85.7% vs. 60%, NAA/Cr: 66.7% vs. 20%, Cho/NAA: 81.1% vs. 33.3%), respectively. This sensitivity was even higher than the ADC index, whereas in the present study, such a concept was not inferred, and no significant difference was observed in relation to the grade of the disease and the level of sensitivity and specificity [40].

The limitations of our study include the short follow-up period (6 months) and the inability to evaluate pathological results after treatment. Moreover, owing to clinical limitations (patient migration and death, low-quality images due to artifacts, etc.), the sample size in the subgroups was small.

## Conclusion

Based on these results, both diffusion and metabolic imaging are suitable for predicting the response to treatment and PPN evaluation. DWI/ADC with high sensitivity and specificity and changes in ADC and Cho/Cr and Cho/NAA ratios can be used to differentiate between patients with PPN from PN.

## Data Availability

Due to the privacy of patients and ethical issues, it is not possible to publish data but is available from the corresponding author upon reasonable request.

## Abbreviations

RT: Radiotherapy
CRT: Chemo-radiotherapy
MRI: Magnetic resonance imaging
MRSI: Magnetic resonance spectroscopy imaging
T2wI: T2-weighted
T1wI: T1-weighted
ADC: Apparent diffusion coefficient
Cho: Choline
Cr: Creatine
NAA: N-acetyl aspartate
Epn: Ependymoma
Asr: Astrocytoma
Oli: Oligodendroglia
Asr: Astrocytoma
RANO: Response assessment in neuro-oncology
TMZ: Temozolomide
NPN: Non-progression
PN: Progression
CELV: Contrast-enhanced lesion volume
NELV: Non-enhanced lesion volume
NTV: Necrotic tumor volume
fx: Fraction
FOV: Field of view
ROI: Region of interest
ms: Milliseconds
M: mean
m: month
